# Se-methylselenocysteine inhibits phosphatidylinositol 3-kinase activity of mouse mammary epithelial tumor cells *in vitro*

**DOI:** 10.1186/bcr1276

**Published:** 2005-07-06

**Authors:** Emmanual Unni, Dimpy Koul, Wai-Kwan Alfred Yung, Raghu Sinha

**Affiliations:** 1 Medicine Endocrinology, Baylor College of Medicine, Houston, Texas, USA; 2 Department of Neuro-Oncology, University of Texas MD Anderson Cancer Center, Houston, Texas, USA; 3 Department of Biochemistry and Molecular Biology, Penn State College of Medicine, Hershey, Pennsylvania, USA

## Abstract

**Introduction:**

Se-methylselenocysteine (MSC), a naturally occurring selenium compound, is a promising chemopreventive agent against *in vivo *and *in vitro *models of carcinogen-induced mouse and rat mammary tumorigenesis. We have demonstrated previously that MSC induces apoptosis after a cell growth arrest in S phase in a mouse mammary epithelial tumor cell model (TM6 cells) *in vitro*. The present study was designed to examine the involvement of the phosphatidylinositol 3-kinase (PI3-K) pathway in TM6 tumor model *in vitro *after treatment with MSC.

**Methods:**

Synchronized TM6 cells treated with MSC and collected at different time points were examined for PI3-K activity and Akt phosphorylation along with phosphorylations of Raf, MAP kinase/ERK kinase (MEK), extracellular signal-related kinase (ERK) and p38 mitogen-activated protein kinase (MAPK). The growth inhibition was determined with a [^3^H]thymidine incorporation assay. Immunoblotting and a kinase assay were used to examine the molecules of the survival pathway.

**Results:**

PI3-K activity was inhibited by MSC followed by dephosphorylation of Akt. The phosphorylation of p38 MAPK was also downregulated after these cells were treated with MSC. In parallel experiments MSC inhibited the Raf–MEK–ERK signaling pathway.

**Conclusion:**

These studies suggest that MSC blocks multiple signaling pathways in mouse mammary tumor cells. MSC inhibits cell growth by inhibiting the activity of PI3-K and its downstream effector molecules in mouse mammary tumor cells *in vitro*.

## Introduction

Several organic and inorganic selenium compounds have been reported to be effective chemopreventive agents against multiple models of mammary tumorigenesis in both the mouse and the rat [1-5]. Selenium compounds have been shown to exert marked stage specificity, especially in preneoplastic mammary lesions, but neither normal mammary gland development nor existing mammary tumor growth was affected by selenium supplemental status [1,6,7]. Although the precise mechanisms by which selenium compounds inhibit mammary tumorigenesis are not well understood, there is evidence that the inorganic and organic selenium compounds act through different pathways [8-10]. Selenium compounds have been reported to affect numerous cellular events and molecular pathways leading to apoptosis. Molecular targets for various natural and synthetic organoselenium compounds have been reviewed [11-15].

Selenite, a widely used inorganic selenium compound, is considered cytotoxic and causes single-stranded DNA breaks and also other non-specific effects [16]. In contrast, Se-methylselenocysteine (MSC) is a less toxic organic selenium compound occurring naturally. It is the major form of selenium compound in selenium-enriched garlic, onions and broccoli [17]. In the mammary tumor model, MSC is more efficacious than the most extensively studied selenoamino acids in animal models [15,18]. Furthermore, MSC inhibits cell growth in several mouse mammary tumor cell lines [19,20] and human breast cancer cell lines [21]. We and other investigators have shown that this inhibition of cell growth is mediated through the induction of apoptosis *in vitro *[20-22] and *in vivo *[23-25]. Using a synchronized mouse mammary cell line TM6, we have shown previously that MSC inhibits DNA synthesis, followed by the arrest of cells in S phase [19]. This block is associated with decreased cdk2 kinase activity [19] and altered cdk2 phosphorylation [26]. In addition, treatment of cells with MSC decreases PKC activity and increases *gadd *(*34*, *45 *and *153*) gene expression in a time-dependent manner [26]. Furthermore, using the same model system, we also reported increased caspase-3, caspase-6 and caspase-8 activities, leading to apoptosis in the MSC-treated TM6 cells in a synchronized model [22].

The effect of MSC on mammary survival pathways is not well understood. One of the earliest responses of starved cells that are exposed to extracellular stimulation with growth factors including serum is the simultaneous activation of both the Raf–MAP kinase/ERK kinase–extracellular signal-related kinase (Raf–MEK–ERK) and phosphatidylinositol 3-kinase (PI3-K)–Akt pathways [27,28]. Activation of Raf can lead to opposing cellular responses such as proliferation, growth arrest, apoptosis or differentiation, depending on the duration and strength of the external stimulation and on the cell type [29]. There is a lack of published data on the effect of selenium on Raf in mammary tumors. PI3-K regulates diverse cellular functions such as growth, survival and malignant transformation through its multiple enzymatic functions, namely lipid kinase and protein kinase activities [30,31], and acts either synergistically with the Raf pathway [32] or in opposition to it [33]. There are few reports demonstrating effects of selenium on PI3-K, but the effect of MSC on PI3-K activity has not been reported previously. One of the possible anti-apoptotic effects of PI3-K is brought about by the phosphorylation of Akt, which in turn can cross-talk with Raf by phosphorylating it at a highly conserved serine residue (Ser259) in its regulatory domain and inhibiting the activation of the Raf–MEK–ERK pathway. The effects of selenium on Akt are limited and the results vary depending on the form (whether inorganic or organic) and on cell type. For the present investigation we examined the effects of MSC on the components of the PI3-K–Akt and Raf–MEK–ERK pathways to improve our understanding of the mechanisms of growth inhibition in the synchronized TM6 mouse mammary tumor cell line.

## Materials and methods

### Cell culture and treatment with MSC

**Figure 1:**
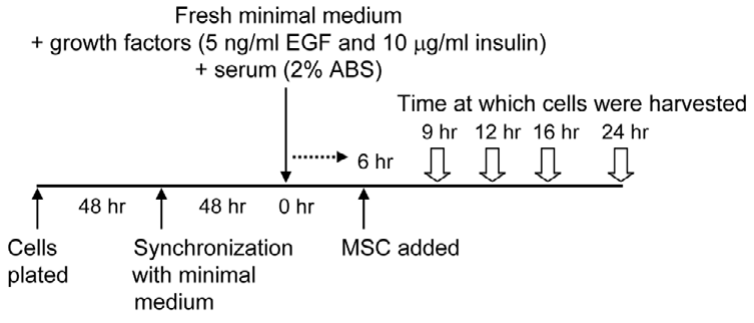
General scheme for synchronization and treatment of TM6 cells with Se-methylselenocysteine (MSC) including the collection times. The TM6 cells were plated at a density of 6.6 × 10^3 ^cells/cm^2 ^in either 100 mm dishes or six-well plates. After 48 hours of growth the cells were starved in DMEM/F12 medium without growth factors and serum (minimal medium) for a further 48 hours. The cells were released from starvation with DMEM/F12 medium containing growth factors (5 ng/ml epidermal growth factor (EGF) and 10 μg/ml insulin) and serum (2% adult bovine serum). After a further 6 hours MSC was added at a final concentration of 50 to 400 μM (depending upon the experiment) to one set of cells. Untreated cells served as controls. The cells were collected after starvation (0 hours), then at 6 (before the addition of MSC), 9, 12, 16 and 24 hours time-points.

The TM6 tumor cell line was originally derived from the non-tumorigenic COMMA-D mouse mammary epithelial cell line [34]. TM6 tumor cells generate alveolar mammary tumors in Balb/c mice when injected into the fat pads. These tumors are p53 mutant and are predicted to be estrogen independent. TM6 cells were cultured routinely in DMEM/F-12 medium containing growth factors (5 ng/ml epidermal growth factor, 10 μg/ml insulin), serum (2% adult bovine serum) and 1 × antibiotic–antimycotic solution (Invitrogen Corporation, Carlsbad, CA, USA) in the presence of 5% CO_2 _in air at 37°C [19]. In brief, the cells were plated at a density of 6.6 × 10^3 ^cells/cm^2 ^in either 100 mm dishes or six-well plates. After growth for 48 hours (Fig. 1) the cells were starved in DMEM/F12 medium without growth factors and serum (minimal medium) for a further 48 hours. The cells were then released from starvation with DMEM/F12 medium containing growth factors and serum. After a further 6 hours, MSC (Sigma, St Louis, MO, USA) was added at a final concentration of 100 μM (unless otherwise mentioned) to one set of cells. Cells were collected after starvation (0 hours), then at 6 (before the addition of MSC), 9, 12, 16 and 24 hours. These times reflect the points at which cells were stimulated with growth factors and serum after starvation, minus 6 hours of treatment time with MSC as described previously [19].

### MSC pretreatment

To study the effect of MSC on the native and phosphorylated Akt, Raf and MEK signals that arise immediately after the addition of medium containing growth factors and serum to starved cells, the cells were synchronized in minimal medium for at least 24 hours. MSC was then added (in minimal medium) for the stipulated time points. The cells were stimulated with fresh DMEM/F12 medium containing growth factors and serum in the continued presence of MSC and were harvested 1 hour later. In these experiments, the time refers to the point at which the cells were pretreated with MSC before the stimulation.

### Incorporation of [^3^H]thymidine

Synchronized TM6 cells grown in 12-well plates (2.5 × 10^4 ^cells per well) were treated with 50 μM MSC for various durations and pulsed for 1 hour with 1 μCi of [^3^H]thymidine (MP Biomedicals, Irvine, CA, USA) per well. After three washings with Tris-buffered saline, the cells were treated with 10% trichloroacetic acid for 5 min followed by two washes with trichloroacetic acid. The incorporation of [^3^H]thymidine was determined by counting the vials in a liquid-scintillation counter. The assay was performed in triplicate for all time points [19].

### Antibodies

Polyclonal anti-(phospho-Akt (Ser473)), anti-Akt, anti-(phospho-Raf), anti-(phospho-MEK), anti-(phospho-ERK (p44/p42)), anti-(phospho-p38 MAPK) and horseradish peroxidase (HRP)-conjugated anti-rabbit antibody were purchased from New England Biolabs (Beverly, MA, USA). Monoclonal anti-PTEN, anti-actin and HRP-conjugated anti-goat antibody were purchased from Santa Cruz Biotechnology (Santa Cruz, CA, USA). Anti-(PI3-K (p85)) antibody was purchased from Upstate (Lake Placid, NY, USA).

### Isolation of protein and immunoblotting

Cell pellets collected after being washed with cold PBS were lysed for 30 min in a buffer containing 20 mM Tris-HCl (pH 7.5), 150 mM NaCl, 1 mM EDTA, 1 mM EGTA, 1% Triton X-100, 2.5 mM sodium pyrophosphate, 1 mM β-glycerophosphate, 1 mM Na_3_VO_4_, 1 μg/ml leupeptin and 1 mM phenylmethylsulphonyl fluoride on ice. The post-mitochondrial supernatants were collected after centrifugation at 8,000 *g *for 10 min and were measured for total protein content with a BCA™ Protein Assay Kit (Pierce, Rockford, IL, USA). Equal amounts of protein were loaded for a given western blot analysis. A range of 20 to 50 μg of protein was loaded in each lane as indicated in the respective figure legends. Immunoblot analysis was performed as described previously [19]. The signals were detected by enhanced chemiluminescence (Amersham Biosciences Corp, Piscataway, NJ, USA) and quantified with the ImageQuant software (Molecular Dynamics, Sunnyvale, CA, USA). The protein loading on gels was normalized to that of actin.

### PI3-K activity

PI3-K activity was measured with the method described by Truitt and colleagues [35]. The cell pellets were lysed in solubilization buffer containing 50 mM HEPES (pH 7.0), 150 mM NaCl, 1 mM EGTA, 10 mM NaF, 10 mM sodium pyrophosphate, 10% glycerol, 1% Triton X-100, 1 mM Na_3_VO_4_, 1 μM pepstatin, 10 μg/ml aprotinin, 5 mM iodoacetic acid and 2 μg/ml leupeptin. Cell extracts (500 μg) were then incubated for 2 hours with 4 μl of anti-PI3-K at 4°C and for a further 2 hours with 50 μl of Protein A–Sepharose beads (Amersham Biosciences Corp). After centrifugation, the immunoprecipitates were washed sequentially as follows: first, three times with PBS containing 1% Triton X-100 and 100 μM Na_3_VO_4_; second, twice with 100 mM Tris-HCl (pH 7.6), 0.5 M LiCl and 100 μM Na_3_VO_4_; third, twice with 100 mM Tris-HCl (pH 7.6), 100 mM NaCl, 1 mM EDTA and 100 μM Na_3_VO_4_; and fourth, twice with 20 mM HEPES (pH 7.5), 50 mM NaCl, 1 mM EDTA, 30 mM sodium pyrophosphate, 200 μM Na_3_VO_4_, 0.03% Triton X-100 and 1 mM phenylmethylsulphonyl fluoride.

The washed immunoprecipitates were resuspended in 30 μl of kinase buffer containing 33.3 mM Tris-HCl (pH 7.6), 125 mM NaCl, 16.6 mM MgCl_2_, 164.3 mM adenosine and 16.6 μM ATP. To this mix, 30 μCi of [γ-^32^P]ATP (1 mCi/100 μl), 7 μl of water and 20 μg of phosphatidylinositol 4-monophosphate prepared in 10 μl of 20 mM HEPES (pH 7.5) was added. The reaction was performed at room temperature on a rotary mixer for 30 min. After the addition of 100 μl of 1 M HCl to stop the reaction, the phosphorylated substrate was extracted with 600 μl of chloroform : methanol (1:1). The organic phase was then separated by centrifugation at 3,000 r.p.m. for 5 min, re-extracted with 200 μl of deionized water and dried by centrifugation under vacuum. The lipid was redissolved in 20 μl of chloroform : methanol (1:1) mixture. The radiolabeled phosphatidylinositol phosphate was resolved on silica gel G-60 thin-layer chromatography plates by chromatography for 3 hours in a solvent system of chloroform : methanol : ammonium hydroxide : water (60:47:2:11.3) and was revealed by autoradiography.

## Results

**Figure 2:**
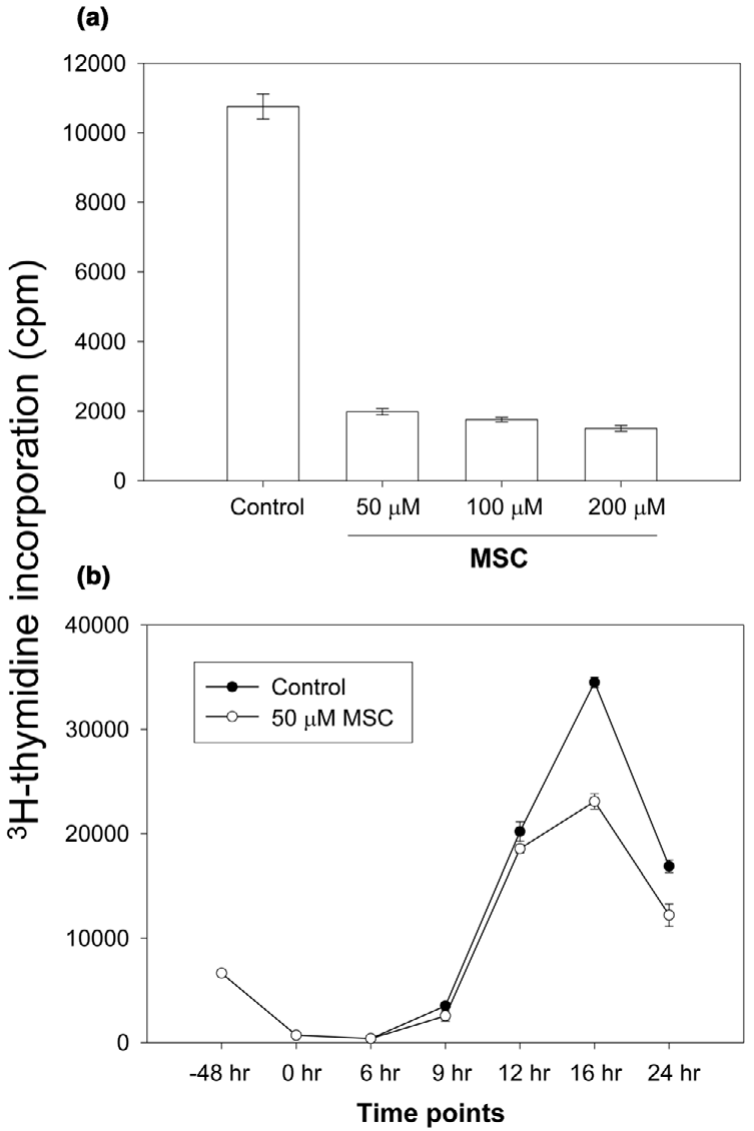
[^3^H]Thymidine incorporation into TM6 cells after Se-methylselenocysteine (MSC) treatment. **(a) **Asynchronous TM6 cells were grown for 24 hours and treated with various concentrations of MSC for 48 hours to determine the optimum dose for treating synchronized cells. **(b) **Synchronized TM6 cells were treated with 50 μM MSC at 6 hours and the DNA synthesis was measured by [^3^H]thymidine incorporation at the indicated time points as described in the Materials and methods section. Data are presented as means ± SEM for three observations at each given time point. MSC at 50 μM showed a greater ability to block DNA synthesis in asynchronous TM6 cells, mainly because of the extended time of treatment.

Treatment with MSC inhibited DNA synthesis in both asynchronous (Fig. 2a) and synchronized (Fig. 2b) TM6 mouse mammary epithelial tumor cells, as measured by [^3^H]thymidine incorporation. The untreated control cells incorporated maximum [^3^H]thymidine at 16 hours when most of the cells are in S phase, as reported previously [19], whereas DNA synthesis in cells treated with 50 μM MSC was inhibited by 33% at this time point. The same dose of MSC suppressed [^3^H]thymidine incorporation to a greater degree in asynchronous cells; this was mainly due to the longer treatment period, 48 hours.

**Figure 3:**
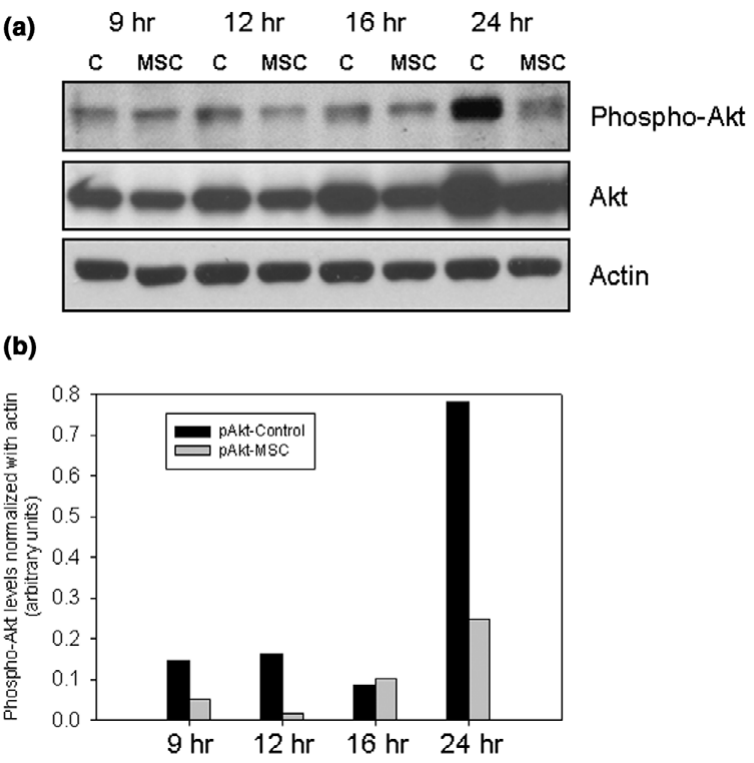
Effect of Se-methylselenocysteine (MSC) on Akt. Synchronized TM6 cells were treated with 50 μM MSC as described in Fig. 1. For each time point three 100 mm dishes were treated with MSC and pooled for protein content. Equal amounts of protein lysates were loaded on each lane (50 μg) for each time point. **(a) **Immunoblots were probed with anti-Akt, anti-phospho-Akt (Ser473) and anti-actin as described in the Materials and methods section. The phosphorylation of Akt occurring at 24 hours in control cells was inhibited in the MSC-treated cells. **(b) **The levels of phospho-Akt in control and MSC-treated cells were normalized with respective actin contents and plotted against various time points.

MSC induces apoptosis in mammary epithelial tumor cells [19,20] and we have documented that caspase-3 activity is enhanced in MSC-treated cells at 24 hours [22]. Because the activation of caspase-3 is a late event in the progression of apoptosis, we examined the phosphorylation of Akt, which is one of the early key signals controlling proliferation and/or apoptosis. The expression of Akt protein remained unchanged in MSC-treated and untreated control cells until 24 hours (Fig. 3). However, at 24 hours there was an increase in Akt phosphorylation in the control cells, and a 68% decrease in MSC-treated cells. This decrease in phospho-Akt was not due to a decline in the native Akt levels.

**Figure 4:**
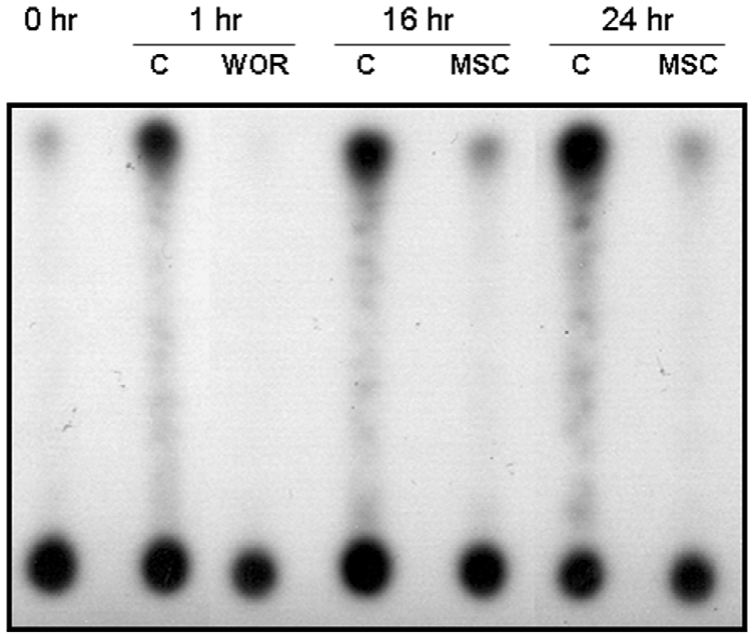
Effect of Se-methylselenocysteine (MSC) on phosphatidylinositol 3-kinase (PI3-K) activity in TM6 cells. Synchronized TM6 cells were treated with 100 μM MSC at 6 hours as described in Fig. 1. Another set of TM6 cells were pretreated with 1 μM wortmannin (WOR) for 30 min before the 1 hour stimulation with fresh DMEM/F12 medium containing growth factors (5 ng/ml epidermal growth factor and 10 μg/ml insulin) and serum (2% adult bovine serum). PI3-K activity was performed on 500 μg of protein lysates as described in the Materials and methods section. The kinase activity in the control cells increased within 1 hour of stimulation, and was strongly inhibited by WOR. The PI3-K activity in MSC-treated cells at 16 and 24 hours were drastically lowered compared with that of the control cells. The data are a representative of experiments performed in triplicate for each time point.

Since PI3-K is an upstream target of Akt, we wished to determine whether this decrease in phospho-Akt levels in MSC-treated cells was in fact due to a lower PI3-K activity. For measuring the activity, PI3-K from control and MSC-treated cells (16 hours and 24 hours) was immunoprecipitated with anti-p85 antibody and assayed for its ability to phosphorylate phosphatidylinositol 4-monophosphate. In the TM6 synchronized model, PI3-K activity increased within 1 hour of stimulation with serum (Fig. 4); this was blocked by 1 μM wortmannin (PI3-K inhibitor). There was a 73% and 84% decrease in PI3-K activity in MSC-treated cells at 16 and 24 hours, respectively, in comparison with the control cells.

**Figure 5:**
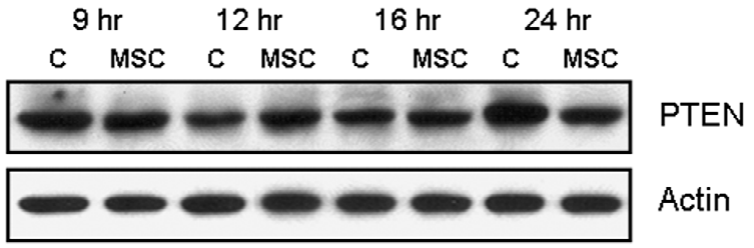
Effect of Se-methylselenocysteine (MSC) on PTEN levels in TM6 cells. Synchronized TM6 cells were treated with 50 μM MSC as described in Fig. 1. An equal amount of protein lysates (50 μg) was loaded on each lane. Immunoblots were probed with anti-PTEN and anti-actin as described in the Materials and methods section. The protein levels of PTEN in the control cells were not significantly different from that of MSC-treated cells at various time points.

Because PI3-K is inactivated by the lipid phosphatase PTEN (MMAC1), we further examined whether the decrease in PI3-K activity was due to an increase in PTEN levels. The levels of PTEN were determined at different time points by immunoblotting (Fig. 5); no appreciable differences were observed between MSC-treated and control cells up to 24 hours.

**Figure 6:**
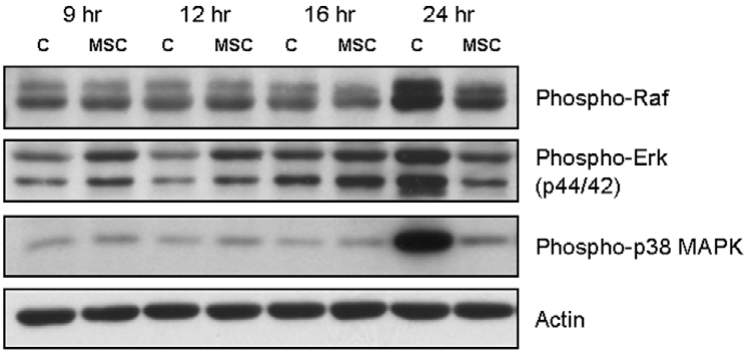
Effect of Se-methylselenocysteine (MSC) on phospho-Raf, phospho-ERK, phosphorylated p38 mitogen-activated protein kinase (phospho-p38 MAPK) levels in TM6 cells. TM6 cells were synchronized and treated with 50 μM MSC as described in Fig. 1. Equal amounts of TM6 lysates (50 μg of protein) were loaded on each lane. Immunoblots were probed with anti-phospho-Raf, anti-phospho-ERK, anti-phospho-p38 MAPK and anti-actin as described in the Materials and methods section. Levels of the phosphoproteins remained unchanged at 9, 12 and 16 hours but at 24 hours the phosphorylated proteins decreased in MSC-treated cells.

Treatment with MSC of TM6 cells at 24 hours inhibited both Akt phosphorylation (Fig. 3) and PI3-K activity (Fig. 4). The lowered PI3-K activity could be due either to an effect of MSC on the enzyme activity or to the inhibition of an upstream event, such as Ras activation. To dissect the two possibilities we examined the two independent downstream parallel pathways that were activated by Ras: first, the activation of Raf by Ras and its downstream targets MEK and ERK, and second, the activation of PI3-K and its downstream targets Akt and p38 mitogen-activated protein kinase (MAPK). We speculated that if MSC inhibits Ras along with the decrease in phospho-Akt levels, which we had observed at 24 hours, the phosphorylation of p38 MAPK or ERK should also decline. Fig. 6 shows the phosphorylated state of Raf in MSC-treated and untreated cells at different time points. The levels remained unchanged in both the samples at 9, 12 and 16 hours. At 24 hours the phospho-Raf levels were 58% lower in MSC-treated cells. A similar pattern of decreased phosphorylation was observed for phospho-Erk (p44/42) when MSC-treated and control cells were compared at different time points. The phosphorylation pattern of phospho-p38 MAPK, a downstream target of Akt, mimicked the pattern of phospho-Akt levels in MSC-treated versus control cells. There was no difference in the phospho-p38 MAPK levels in MSC-treated and control cells until 24 hours. However, the levels of phospho-p38 MAPK increased at 24 hours in control cells and were inhibited more than threefold in MSC-treated cells. The levels of native Akt, ERK, p38 MAPK and Raf proteins did not change with treatment with MSC (data not shown).

**Figure 7:**
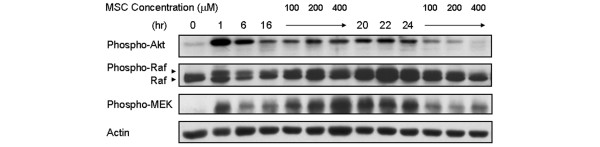
Effect of Se-methylselenocysteine (MSC) on phospho-Akt, phospho-Raf and phospho-MEK in TM6 cells. The TM6 cells were synchronized in minimal medium as described in Fig. 1, but only for 24 hours. Then the MSC (100 to 400 μM) was added (in minimal medium) for 16 and 24 hours. The cells were stimulated with fresh DMEM/F12 containing growth factors (5 ng/ml epidermal growth factor and 10 μg/ml insulin) and serum (2% adult bovine serum) at the indicated time points for 1 hour. Equal amounts of lysates (20 μg of protein) were loaded on each lane for each time point. Immunoblots were probed with anti-phospho-Akt, anti-phospho-Raf, anti-phospho-MEK and anti-actin antibodies as described in the Materials and methods section. Both the Akt and Raf were phosphorylated within 1 hour of stimulation with growth factor and serum. At 16 hours a dose of 400 μM MSC failed to inhibit the phosphorylation of Akt or Raf and the downstream effector MEK. However, at 24 hours MSC was able to inhibit phosphorylation of all three proteins in a dose-dependent manner.

To distinguish between the tolerance of MSC concentrations and their effects in signaling, components of both the Raf and Akt pathways, namely phosphoprotein levels of Akt, Raf and MEK, were analyzed in TM6 cells synchronized in minimal medium for 24 hours and then treated with different doses of MSC in minimal medium for 16 and 24 hours before stimulation with growth factors and serum. As expected, all three proteins were phosphorylated within 1 hour of stimulation (Fig. 7). At 16 hours, even at 400 μM MSC, the phosphorylated protein levels of Akt and Raf were comparable to that of the control. However, at 24 hours their levels decreased with increasing concentrations of MSC. The native Akt and MEK levels did not show an appreciable change at all time points (data not shown); the native Raf protein expression did not change either during this experiment. The immunoblot in Fig. 6 also demonstrates that at 24 hours the levels of these phosphoproteins started to increase in the control cells, indicating the start of a second wave of stimulation.

**Figure 8:**
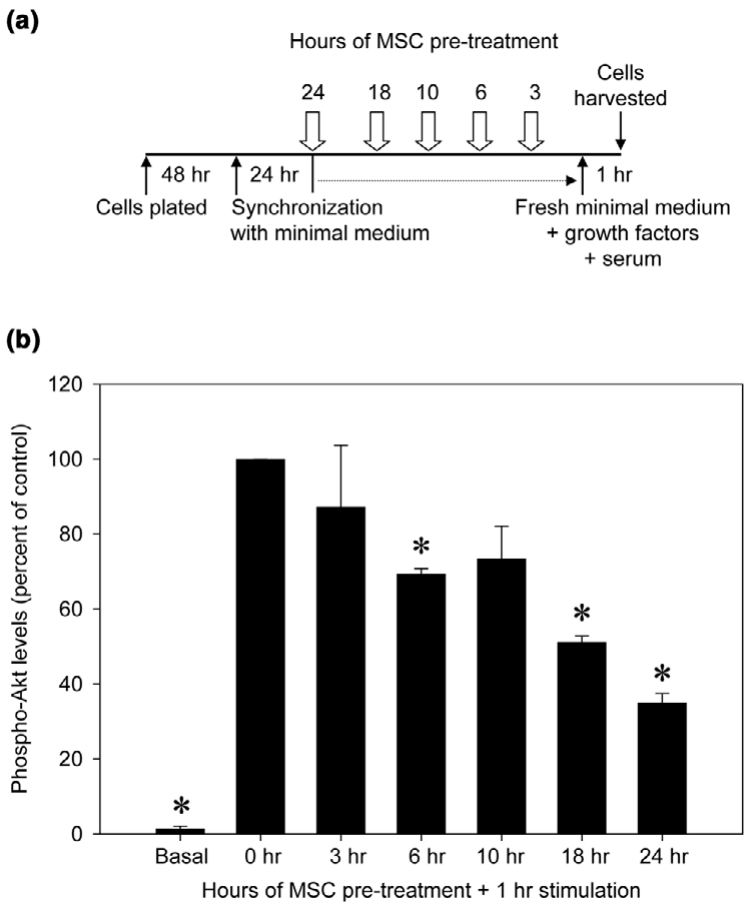
Effect of Se-methylselenocysteine (MSC) on Akt phosphorylation. **(a) **Scheme for pretreatment of TM6 cells with MSC. Cells were synchronized in minimal medium for 24 hours. Cells were then exposed to 100 μM MSC for 3, 6, 10, 18 and 24 hours in minimal medium before being stimulated with fresh DMEM/F12 medium containing growth factors (5 ng/ml epidermal growth factor and 10 μg/ml insulin) and serum (2% adult bovine serum) for 1 hour. **(b) **Effect of pretreatment of MSC on Akt phosphorylation in TM6 cells. Equal amounts of lysates (30 μg of protein) were loaded on each lane for each time point. After electrophoresis the immunoblots were probed with anti-phospho-Akt (Ser473) antibody as described in the Materials and methods section, and the levels were measured with Molecular Dynamics software. Each bar represents levels in TM6 tumor cells treated with MSC in three different wells. *P < 0.05 compared with 0 hours.

To examine whether MSC needs to be metabolized to have an effect on the phosphorylation of Akt, cells were synchronized with minimal medium for 24 hours and were subsequently treated with 100 μM MSC for various periods (0 to 24 hours), stimulated with growth factors and serum for 1 hour and examined for Akt phosphorylation (Fig. 8a). Pretreatment of the cells with MSC for 10 hours, equivalent to the cells collected at 16 hours in the previous scheme of experiments (Fig. 1), Akt phosphorylation was inhibited by only 26% (Fig. 8b). After 18 and 24 hours' pretreatment of TM6 cells with MSC, the inhibition in phospho-Akt levels was 49% and 65%, respectively, and was significant (*P*<0.05) when compared with untreated cells.

## Discussion

The results presented here demonstrate that MSC inhibits PI3-K activity and subsequently inactivates Akt *in vitro*. This is a significant observation in establishing one of the mechanisms by which MSC inhibits mouse mammary epithelial cell growth *in vitro*.

Previously we had reported that TM6 cells treated with MSC are delayed in S phase at about 24 hours [19,26]. In the present set of experiments the differences in Akt phosphorylation between MSC-treated and untreated control cells occur at about 24 hours. This observation was not clear because Akt phosphorylation is an immediate event, occurring within 1 hour of stimulation with growth factors and serum. Various possibilities exist: first, inhibition of Akt phosphorylation in MSC-treated cells beginning at 24 hours might require the cells to be delayed in S phase; second, there might be a requirement for MSC to be metabolized into an active molecule such as methylselenol [36] that causes inhibition; or third, there might be a slow diffusion of MSC into the cells. We have shown that MSC enters the TM6 cells within 30 min of treatment and can inhibit DNA synthesis in these cells 3 hours later [22], thus excluding the probability of slower diffusion into the cells.

To address the first two of these alternatives, different strategies were designed in TM6 cells. In the first set of experiments (scheme outlined in Fig. 1), the cells were allowed to cycle after stimulation with growth factors and serum, and MSC was added 6 hours later. In these experiments, events leading to Akt phosphorylation had already taken place before the addition of MSC. By 16 hours, although PI3-K activity was inhibited in the MSC-treated cells, the phospho-Akt levels remained unchanged in both the control and MSC-treated cells. In the TM6 synchronization model we noted that the Akt phosphorylation is stimulated again at a later time point in the cell cycle. The occurrence of this 'second wave of stimulation' is quite evident from an elevated level of phospho-p38 MAPK at 24 hours in control cells. This stimulation actually appeared at 22 hours (data not shown) in TM6 cells when examined closely. PI3-K activity was inhibited at about 16 hours, and thus its effect on Akt phosphorylation occurs only with the second wave of stimulation. This could explain why phospho-Akt levels were the same in both MSC-treated and untreated control cells at 16 hours even though the PI3-K activity was inhibited in the MSC-treated cells.

Second, the fact that PI3-K activity is inhibited earlier than Akt-phosphorylation supports the hypothesis that the upstream target of MSC-induced growth inhibition is PI3-K. When the cells were pretreated with MSC and then stimulated with growth factors and serum, there was a gradual inhibition of Akt phosphorylation. Most of the cells during this synchronization state would be predicted to be in G1 phase during this time [19], so the possibility that factors causing a delay in S phase might result in a decreased phosphorylation of Akt can be excluded.

**Figure 9:**
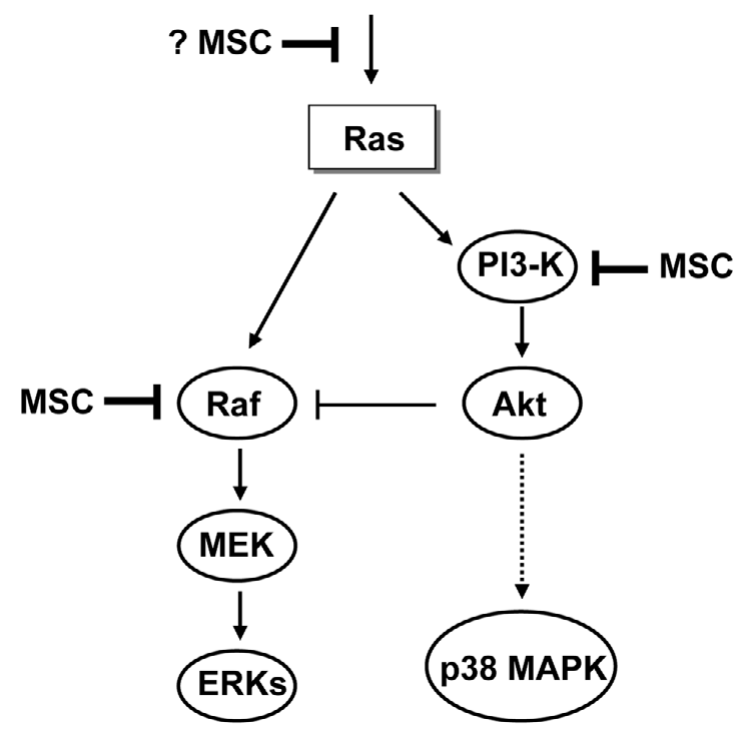
Possible sites of Se-methylselenocysteine (MSC) interaction with components of the Ras–phosphatidylinositol 3-kinase–Akt (Ras–PI3-K–Akt) and Raf–MAP kinase/ERK kinase–ERK (Raf–MEK–ERK) pathways in TM6 mouse mammary tumor cells. MAPK, mitogen-activated protein kinase.

The probable reason that the differences in the Akt phosphorylation are not observed until 24 hours is that MSC might need to be metabolized to methylselenol before it can effectively inactivate Akt. MSC can be metabolized into methylselenol, which could be dimethylated and trimethylated to dimethylselenide or trimethylselenonium respectively [37]. Other organoselenium compounds such as dimethylselenoxide and selenobetaine methyl ether can be metabolized to dimethylselenide and trimethylselenonium without the formation of methylselenol and do not have anticancer activity. It has therefore been suggested that methylselenol is the active proximal molecule of MSC [37]. MSC is capable of generating methylselenol endogenously through the action of β-lyase or related lyases [38]. As the cells in culture have low levels of β-lyase, it leads to the inefficient conversion of MSC to methylselenol [23,39,40], and so we used higher doses of MSC (100 to 400 μM) in some of our experiments. Several current studies have looked at an alternative methylselenol generator, methylseleninic acid, a compound that represents a simplified version of MSC without the amino acid moiety, thereby obviating the need for β-lyase action. There are a few reports indicating the differential effect of selenium compounds on Akt in vascular endothelial [41], prostate [42], mammary [43] and oral [44] cancer cells depending on the form of selenium. On the basis of our present results the speculated sites of MSC interaction with components of Ras–PI3-K–Akt pathway and Raf–MEK–ERK pathway are illustrated in Fig. 9.

Akt interacts with Raf and phosphorylates it at Ser259. Furthermore, phosphorylation of Raf by Akt inhibits activation of the Raf–MEK–ERK signaling pathway and has been shown to alter the cellular response in a human breast cancer cell line from cell cycle arrest to proliferation [29]. Our results indicate that this cross-talk between Akt and Raf might be altered by MSC. It has also been reported that Akt is a substrate for caspase and cleaves it into 40 and 44 kDa fragments [45]. We have recently shown that the activities of caspase-3, caspase-6 and caspase-8 are increased at 24 hours of treatment with MSC [22]. The cleaved phospho-Akt proteins were observed at 24 hours in MSC-treated cells. It is unlikely that the decrease in Akt phosphorylation at 24 hours was due to elevated caspase activity because PI3-K was inhibited at 16 hours, before the activation of these caspases could be detected in the cells.

It was recently demonstrated that certain tumor suppressor agents downregulate PI3-K by activating the expression of PTEN/MMAC1, a phosphatase that dephosphorylates phosphatidylinositol 3,4,5-trisphosphate [46]. Although MSC could inhibit PI3-K activity in the present study this inhibition was not due to elevated levels of PTEN.

PI3-K is a heterodimer with a catalytic and a regulatory subunit. The catalytic subunit possesses both lipid kinase and serine–threonine protein kinase activities. PI3-K is activated by the binding of either receptor or non-receptor tyrosine kinases to the regulatory subunit; this complex is directed to the membrane and associates with its phospholipid substrate [47]. Because the lipid kinase activity of PI3-K is inhibited on treatment with MSC before any effect on the phosphorylation of Akt, it would be interesting to examine whether MSC could block the integration of PI3-K to the membrane; this is part of an investigation currently in progress. Another important scenario might be if MSC were shown to interfere with the activity of Ras, because both phospho-Raf and phospho-Akt levels are lowered during treatment with MSC. To perform its function, the active form of Ras (GTP-Ras) must also be anchored to the cellular membrane through a post-translationally added lipophilic (iso) prenyl group [48]. Further studies are required to investigate whether MSC alters the anchoring of Ras and PI3-K into the cell membrane.

## Conclusion

The present studies show that MSC blocks multiple pathways in mouse mammary tumor cells *in vitro*. Decreased PI3-K activity in addition to dephosphorylation of Akt by MSC contributes to the growth inhibition of TM6 mouse mammary epithelial cells. This information, along with the possibility that p38 MAPK is a target for the action of MSC on mammary cells, will provide further evidence of its mechanistic inhibition of mammary growth. These experiments need to be translated into human cell lines and xenograft model systems before this compound can be promoted for clinical trials in humans for breast cancer prevention.

## Abbreviations

DMEM/F12 = Dulbecco's modified Eagle's medium/nutrient mixture F-12 Ham; ERK = extracellular signal-related kinase; HRP = horseradish peroxidase; MAPK = mitogen-activated protein kinase; MSC = Se-methylselenocysteine; PI3-K = phosphatidylinositol 3-kinase; TM6 = mouse mammary epithelial tumor cells.

## Competing interests

The author(s) declare that they have no competing interests.

## Authors' contributions

EU treated cells and performed western blot analyses for the native and phosphorylated proteins. DK was responsible for PI3-K activity conducted in W-KAY's laboratory. RS established the *in vitro *synchronized TM6 model, performed the [^3^H]thymidine incorporation assay and was responsible for overall design, statistical analysis, and supervision of all the experiments. EU and RS contributed in manuscript writing. All authors read and approved the final manuscript.
